# Correction: Li, et al. LncRNA NEAT1 Silenced miR-133b Promotes Migration and Invasion of Breast Cancer Cells. *Int. J. Mol. Sci.* 2019, *20*, 3616

**DOI:** 10.3390/ijms21155414

**Published:** 2020-07-30

**Authors:** Xinping Li, Siwei Deng, Xinyao Pang, Yixiao Song, Shiyu Luo, Liang Jin, Yi Pan

**Affiliations:** State Key Laboratory of Natural Medicines, Jiangsu Key Laboratory of Druggability of Biopharmaceuticals, School of Life Science and Technology, China Pharmaceutical University, 24 Tongjiaxiang Avenue, Nanjing 210004, China; 1822030704@stu.cpu.edu.cn (X.L.); siwei.deng@hotmail.com (S.D.); 2020171097@stu.cpu.edu.cn (X.P.); 2020171119@stu.cpu.edu.cn (Y.S.); 2020172173@stu.cpu.edu.cn (S.L.); ljstemcell@cpu.edu.cn (L.J.)

The author wishes to make the following correction to this paper [1]. The reason for the correction is an error in the transwell images presented in this article. Two transwell images ((Inh-miR-133b+si-NC) and (Inh-miR-133b+si-TIMM17A) of invasion group in old Figure 5D [1]) are incorrect, and for this reason they should be replaced with the correct new figure (Figure 1).

The correct new Figure 1 is shown below:

The above errors were without material impact on the final results and conclusions of our papers. The authors would like to apologize for any inconvenience caused to the readers by these errors.

## Figures and Tables

**Figure 1:**
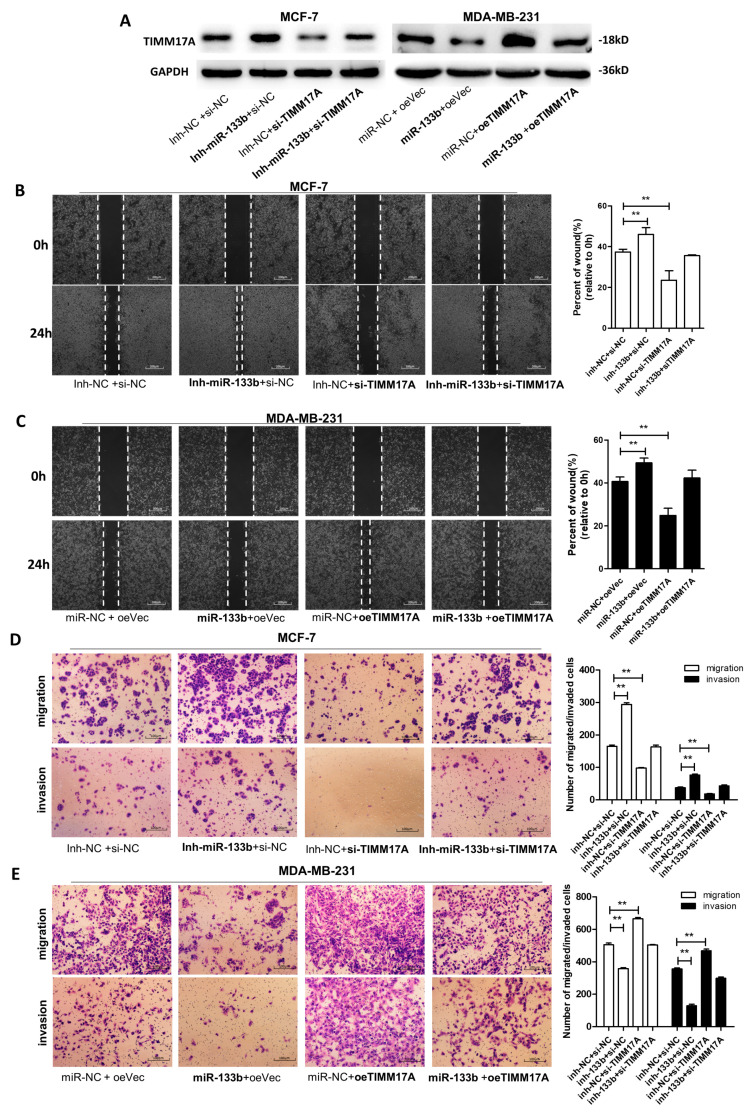
miR-133b promotes breast cancer cell migration and invasion via targeting TIMM17A (**A**) TIMM17A protein levels in MCF-7 cells transfected with inh-NC plus control siRNA(si-NC), inh- miR-133b plus si-NC, inh-NC plus TIMM17A siRNA (si-TIMM17A), or inh- miR-133b plus si-TIMM17A, and in MDA-MB-231 cells transfected with miR-NC plus control vector(oeVec), miR-133b plus oeVec, miR-NC plus TIMM17A vector (oeTIMM17A), or miR-133b plus oeTIMM17A. (**B**,**C**) Migration of MCF-7 cells transfected with inh-NC plus control siRNA(si-NC), inh- miR-133b plus si-NC, inh-NC plus TIMM17A siRNA (si-TIMM17A), or inh- miR-133b plus si-TIMM17A (**B**), and MDA-MB-231 cells transfected with miR-NC plus control vector(oeVec), miR-133b plus oeVec, miR-NC plus TIMM17A vector (oeTIMM17A), or miR-133b plus oeTIMM17A (**C**) detected by wound healing assay. Scale bar, 100 μm. (**D**,**E**) Migration and invasion of MCF-7 cells transfected with inh-NC plus control siRNA(si-NC), inh- miR-133b plus si-NC, inh-NC plus TIMM17A siRNA (si-TIMM17A), or inh- miR-133b plus si-TIMM17A (**D**), and MDA-MB-231 cells transfected with miR-NC plus control vector(oeVec), miR-133b plus oeVec, miR-NC plus TIMM17A vector (oeTIMM17A), or miR-133b plus oeTIMM17A (**E**) detected by transwell migration and invasion assay. Scale bar, 100 μm. ** *p* < 0.01; *** *p* < 0.001.

## References

[1] Li X., Deng S., Pang X., Song Y., Luo S., Jin L., Pan Y. (2019). LncRNA NEAT1 Silenced miR-133b Promotes Migration and Invasion of Breast Cancer Cells. Int. J. Mol. Sci..

