# Supplementary material for: LncRNA NEAT1 Silenced miR-133b Promotes Migration and Invasion of Breast Cancer Cells
Source: Int J Mol Sci. 2019 Jul 24;20(15):3616. doi: 10.3390/ijms20153616 (PMC6695844; doi:10.3390/ijms20153616)
Supplement: Supplementary file 1 [file ijms-20-03616-s001.pdf]

International Journal of
Molecular Sciences

Supplementary Table S1. miR-133b expression and clinicopathological features in 40 patients with breast cancer.

Characteristics	Expression of miR-133b		*p* value*
	Low	High	
**Sex**			
male	20	20	
female	0	0	
**Age**			0.185
≤60	11	15	
>60	9	5	
**Grade**			0.125
Ⅰ/I-II, well-differentiated	4	8	
Ⅱ/II-III, moderately differentiated	9	10	
Ⅲ, poorly differentiated	7	2	
**Tumor histological**			0.001*
Ductal carcinoma in situ	1	10	
Invasive ductal carcinoma	19	10	
**T Classification**			0.062
T1	12	5	
T2	7	11	
T3	1	4	
**N Classification**			0.688
N0	7	6	
N1	3	1	
N2	6	8	
N3	4	5	
**ER status**			0.206
Negative	12	8	
Positive	8	12	
**PR status**			0.288
Negative	16	13	
Positive	4	7	
**HER2 status**			0.736
Negative	6	7	
Positive	14	13	
**Tumor size(cm^3)**			0.109
≤ 6	9	14	
> 6	11	6	
**Lymph node metastasis**			0.008*
Negative	9	17	

Positive	11	3

Median expression level was used as a cutoff to divide the 40 patients into miR-133b low group (n = 20) and miR-133b high group (*n* = 20). Two-sided χ2 test. * $p < 0.05$.

Supplementary Table S2. Sequences of primers used for RT-qPCR and plasmid construction.

Primer Names	Sequences
**Sequences of primers used for RT-qPCR**	
hsa-miR-133b forward	5′-CTCAGCTTTGGTCCCCTTCAAC-3′
hsa-miR-133b reverse	5′-GTGCAGGGTCCGAGGT-3′
U6 forward	5'-ATTGGAACGATACAGAGAAGATT-3'
U6 reverse	5'-GGAACGCTTCACGAATTTG-3'
TIMM17A forward	5'-TTGTGGATGACTGTGGTG-3'
TIMM17A reverse	5'-CCAAAAGGTGAGGAAGGT -3'
GAPDH forward	5'-GAGTCAACGGATTTGGTCGT-3'
GAPDH reverse	5'-TTGATTTTGGAGGGATCTCG-3'
NEAT1 forward	5'-TGGCTAGCTCAGGGCTTCAG-3'
NEAT11 reverse	5'-TCTCCTTGCCAAGCTTCCTTC- 3'
**Sequences of primers used for plasmid construction**	
pcDNA3.1-TIMM17A forward	5'-CAGGAATTCTGGAGGAGTACGCGCGAG- 3'
pcDNA3.1-TIMM17A reverse	5'-GACCTCGAGCTACTGATATTGTCGATA- 3'
pcDNA3.1-NEAT1 forward	5'-CGCGGAGAGTTAGCGACAGGGAGGGAT-3'
pcDNA3.1-NEAT1 reverse	5'-TGCTCTCTAATGAGTTTAGAACTCAAAC-3'

Supplementary Table S3. Sequences of miR-133b mimics and inhibitor, and siRNAs.

RNA Names	Sequences
miR-133b mimics	5′-UUUGGUCCCCUUCAACCAGCUA-3′
mimics NC	5′- UUCUCCGAACGUGUCACGUTT-3′
miR-133b inhibitor	5′-UAGCUGGUUGAAGGGGACCAAA-3′
inhibitor NC	5′-CAGUACUUUUGUGUAGUACAA-3′
NEAT1-siRNA-1	5'-GGTCTGTGTGGAAGGAGGAAGGCAG- 3'
NEAT1-siRNA-2	5′-GCCAUCAGCUUUGAAUAAAUU-3′
NEAT1-siRNA-3	5′-GGUGUUAUCAAGUGAAUUAUU-3′
TIMM17A-siRNA-1	5′-GCAUGAUGUUAGUUAAUUACA-3′
TIMM17A-siRNA-2	5′-GGCUAUAAAGAGACAUUUAGC-3′
TIMM17A-siRNA-3	5′-GAUGUUUCAUGCUCAUGUACU-3′

Supplementary Table S4. Antibodies used for western blotting (WB), RNA-binding protein immunoprecipitation (RIP) and flow cytometry (FC).

**Protein**	**Applications**	**Antibody**	**Origin**	**Dilution**	**Molecular Weight**
GAPDH	WB	D16H11, Cell Signaling Technology	Rabbit	1:1000	36 KD
TIMM17A	WB, CHIP	ab126044, Abcam	Rabbit	1:500	18KD
Ago2	RIP	03-110, Merck Millipore	Mouse	1:10	100KD
GFP	RIP	ab290, Abcam	Rabbit	1:20	28KD

Supplementary Table S5. Screening of 111 predicted targets of miR-133b.

No.	Gene Name	Breast Cancer Related	Migration or Invasion Related	Reported 133b-target	Expression in Tumor	Fold Change	*p*-Value	Survival Related	*p*-Value
1	TIMM17A	√	√	×	high	2.20	7.80E-79	√	2.60E-15
2	ELAVL1	√	√	×	high	1.53	9.60E-67	√	0.007
3	NDRG1	√	√	×	high	1.06	1.40E-05	√	2.20E-09
4	SOX4	√	√	×	high	1.75	5.90E-24	×	0.46
5	PFN2	√	√	×	high	1.61	0.00061	×	0.12
6	CTBP2	√	√	×	high	1.38	9.10E-10	×	0.48
7	RAPH1	√	√	×	low	0.86	0.00054		
8	SGMS2	√	√	×	low	0.76	2.00E-08		
9	CRK	√	√	×	low	0.75	2.10E-26		
10	RBPJ	√	√	×	low	0.74	4.70E-27		
11	TCF7	√	√	×	low	0.59	4.90E-16		
12	MEIS1	√	√	×	low	0.53	7.20E-34		
13	GABARAPL1	√	√	×	low	0.37	5.00E-86		
14	SGK1	√	√	×	low	0.37	1.40E-47		
15	USP6	√	√	×	low	0.36	1.10E-30		
16	PFKFB3	√	√	×	low	0.29	3.70		
17	AKAP9	√	√	×		1.13	0.99		
18	NUP153	√	√	×		1.02	0.30		
19	SP3	√	√	×		1.00	0.58		
20	YES1	√	√	×		0.99	0.014		
21	MAML1	√	√	√					
22	FGFR1	√	√	√					
23	MCL1	√	√	√					
24	LASP1	√	√	√					
25	BCL2L2	√	√	√					
26	DUSP1	√	√	√					
27	MEIS2	√	×						
28	UBA2	√	×						
29	BNIP3L	√	×						
30	PREX1	√	×						
31	PTPRD	√	×						
32	POU4F1	√	×						

33	ATP6AP2	√	×
34	FTL	√	×
35	RARB	√	×
36	TRHDE	√	×
37	MAP3K3	√	×
38	PPP2CA	√	×
39	SH3GL2	√	×
40	AFAP1	√	×
41	USP32	√	×
42	SMARCD1	√	×
43	MECOM	√	×
44	RB1CC1	√	×
45	SV2A	√	×
46	SLC7A8	√	×
47	SUMO1	√	×
48	QKI	√	×
49	VAPB	√	×
50	FOXL2	√	×
51	SEPHS2	√	×
52	TBPL1	√	×
53	PPP2CB	√	×
54	CMPK1	√	×
55	TFE3	√	×
56	SESN1	√	×
57	EPHA7	√	×
58	UBE2Q1	√	×
59	PEX5L	×	
60	AFTPH	×	
61	BTBD3	×	
62	PAN3	×	
63	ARFIP2	×	
64	VPS54	×	
65	ZC3H14	×	
66	ADCYAP1	×	
67	ARHGDIA	×	
68	PRRT2	×	
69	JAZF1	×	

70	DOLPP1	×
71	ZNF436	×
72	CCDC117	×
73	FAM117B	×
74	GARNL3	×
75	GPM6A	×
76	EXD2	×
77	GABPB2	×
78	SUPT16H	×
79	STX5	×
80	ANKRD12	×
81	CLTA	×
82	SOBP	×
83	SACM1L	×
84	FBXL2	×
85	MED12L	×
86	TMOD3	×
87	CDK13	×
88	XPO4	×
89	PPFIA3	×
90	ARHGAP12	×
91	ZNF362	×
92	GRM5	×
93	TRAM2	×
94	TMEM167A	×
95	RAP2C	×
96	PTBP2	×
97	SLC6A1	×
98	FBXW11	×
99	GDI2	×
100	RAVER1	×
101	NRIP3	×
102	SEC61B	×
103	SYT1	×
104	MLLT3	×
105	CRTAM	×
106	RBMXL1	×

107	LRRC7	×
108	TFAP2D	×
109	SHISA5	×
110	SNRK	×
111	MTMR4	×

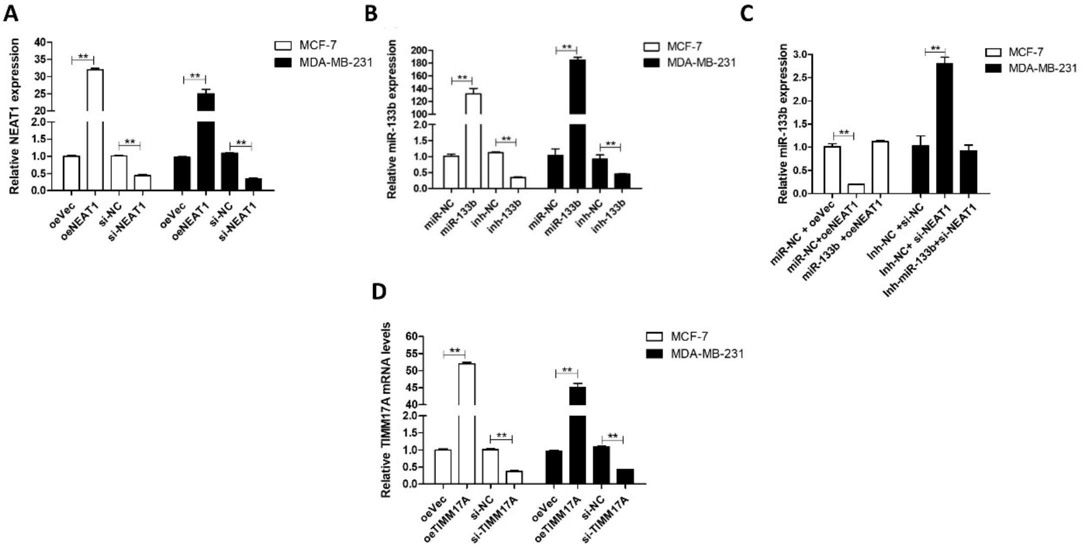

Figure S1. Verification of miR-133b, NEAT1 and TIMM17A overexpression or knockdown efficiencies in breast cancer cells. (**A**) NEAT1 levels in MCF-7 cells and MDA-MB-231 cells transfected with oeVec, oeNEAT1, si-NC or si-NEAT1. (**B**) miR-133b levels in MCF-7 cells and MDA-MB-231 cells transfected with miR-NC, miR-133b, inh-NC or inh-miR-133b. (**C**) miR-133b levels in MCF-7 cells transfected with miR-NC plus oeVec, miR-NC plus oeNEAT1, or miR-133b plus oeNEAT1, and MDA-MB-231 cells transfected with inh-NC plus si-NC, inh-NC plus si-NEAT1, or inh-miR-133b plus si-NEAT1. (**D**) TIMM17A mRNA levels in MCF-7 cells and MDA-MB-231 cells transfected with oeVec, oeTIMM17A, si-NC or si-TIMM17A. $**p < 0.01$; $***p < 0.001$.

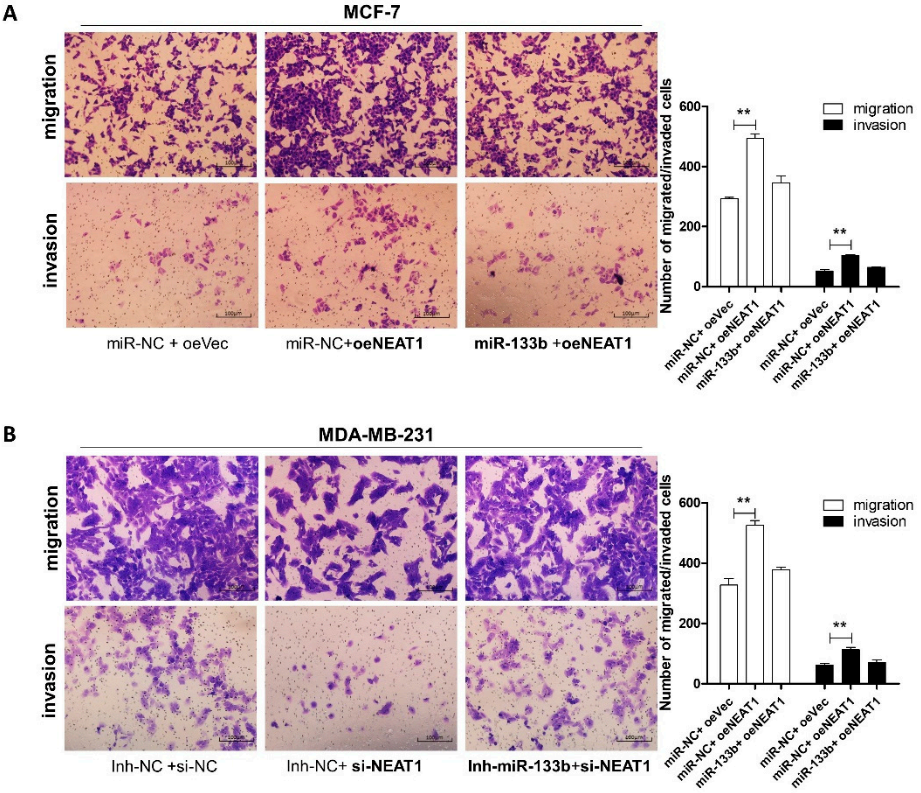

Figure S2. NEAT1 promotes breast cancer cells migration and invasion via silencing miR-133b. (**A**,**B**) Migration and invasion of MCF-7 cells transfected with miR-NC plus oeVec, miR-NC plus oeNEAT1,

or miR-133b plus oeNEAT1 (**A**), and MDA-MB-231 cells transfected with inh-NC plus si-NC, inh-NC plus si-NEAT1, or inh-miR-133b plus si-NEAT1 (**B**) detected by transwell assay. Scale bar, 100 μm.

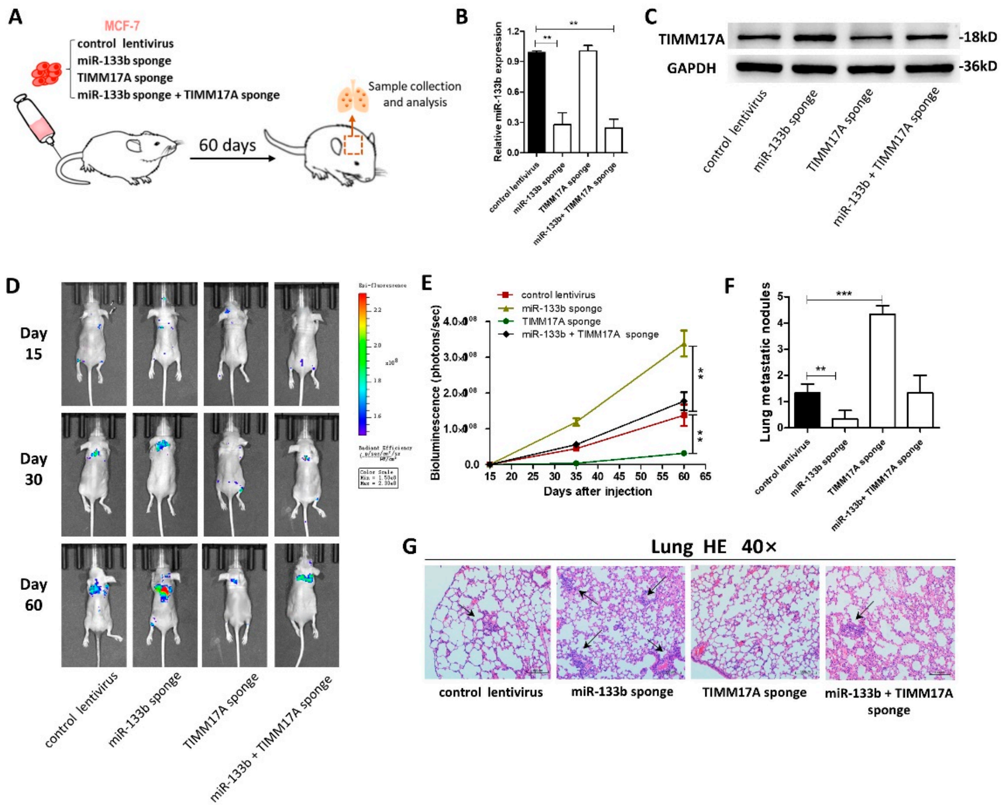

Figure S3. Effects of TIMM17A-targeted miR-133b on the lung colonization of MCF-7 cells xenografts in mice. (**A**) Experimental design: immunocompromised mice were injected through tail vein with MCF-7 cells transfected with either the control lentivirus, miR-133b sponge, TIMM17A sponge, miR-133b sponge plus TIMM17A sponge. (**B**,**C**) miR-133b levels (**B**) and TIMM17A protein levels (**C**) in MCF-7 cells transfected with either the control lentivirus, miR-133b sponge, TIMM17A sponge, miR-133b sponge plus TIMM17A sponge. (**D**,**E**) Representative BLI images (**D**) and quantitative analysis of the fluorescence intensities (**E**) of mice of five groups. The BLI was performed on days 15, 35, and 60 after injection. The intensity of BLI is represented by the color. (**F**,**G**) Numbers of metastatic nodules(**F**) and representative H&E-stained sections of lung tissues isolated from the intravenously injected mice. Black arrows indicate metastatic nodules. Scale bar, 200 μm. **$p < 0.01$; ***$p < 0.001$.
